# Screening and identification of the hub genes in severe acute pancreatitis and sepsis

**DOI:** 10.3389/fmolb.2024.1425143

**Published:** 2024-09-19

**Authors:** Si-Jiu Yang, Yan Luo, Bao-He Chen, Ling-Hui Zhan

**Affiliations:** ^1^ Department of Critical Care Medicine, Zhongshan Hospital of Xiamen University, School of Medicine, Xiamen University, Xiamen, China; ^2^ The School of Clinical Medicine, Fujian Medical University, Fujian, China

**Keywords:** severe acute pancreatitis, sepsis, hub genes, machine learning, immune cell infiltration

## Abstract

**Background:**

Severe acute pancreatitis (SAP) is accompanied with acute onset, rapid progression, and complicated condition. Sepsis is a common complication of SAP with a high mortality rate. This research aimed to identify the shared hub genes and key pathways of SAP and sepsis, and to explore their functions, molecular mechanism, and clinical value.

**Methods:**

We obtained SAP and sepsis datasets from the Gene Expression Omnibus (GEO) database and employed differential expression analysis and weighted gene co-expression network analysis (WGCNA) to identify the shared differentially expressed genes (DEGs). Functional enrichment analysis and protein–protein interaction (PPI) was used on shared DEGs to reveal underlying mechanisms in SAP-associated sepsis. Machine learning methods including random forest (RF), least absolute shrinkage and selection operator (LASSO) and support vector machine recursive feature elimination (SVM-RFE) were adopted for screening hub genes. Then, receiver operating characteristic (ROC) curve and nomogram were applied to evaluate the diagnostic performance. Finally, immune cell infiltration analysis was conducted to go deeply into the immunological landscape of sepsis.

**Result:**

We obtained a total of 123 DEGs through cross analysis between Differential expression analysis and WGCNA important module. The Gene Ontology (GO) analysis uncovered the shared genes exhibited a significant enrichment in regulation of inflammatory response. The Kyoto Encyclopedia of Genes and Genomes (KEGG) pathway analysis revealed that the shared genes were primarily involved in immunoregulation by conducting NOD-like receptor (NLR) signaling pathway. Three machine learning results revealed that two overlapping genes (ARG1, HP) were identified as shared hub genes for SAP and sepsis. The immune infiltration results showed that immune cells played crucial part in the pathogenesis of sepsis and the two hub genes were substantially associated with immune cells, which may be a therapy target.

**Conclusion:**

ARG1 and HP may affect SAP and sepsis by regulating inflammation and immune responses, shedding light on potential future diagnostic and therapeutic approaches for SAP-associated sepsis.

## 1 Introduction

Acute pancreatitis (AP) is a pancreatic inflammatory disorder most usually caused by digestive enzyme activation and self-digestion (Boxhoorn et al., 2020). Most patients in clinical practice experience a self-limiting course; however, approximately 20%–30% of cases will progress to severe acute pancreatitis (SAP), often accompanied by pancreatic infection and peripancreatic necrosis, potentially leading to life-threatening complications (Lee and Papachristou, 2019). Sepsis is one of the common complications of SAP. It is defined as a dysregulated host response to infection characterized by organ dysfunction and has become the main cause of death in SAP patients (Singer et al., 2016; Mifkovic et al., 2006).

Previous studies have shown that SAP can lead to systemic inflammation and immune dysfunction, paving the way for the development of sepsis (Beger and Rau, 2007; Kylänpää et al., 2012). The initial pancreatic injury can trigger an inflammatory response, leading to a waterfall effect and systemic inflammatory response syndrome (SIRS) (Kylänpää et al., 2012). Subsequently, the response shifted from pro-inflammatory to anti-inflammatory. During this transformation process, the patient’s intestinal barrier may be disrupted, leading to bacterial translocation and pancreatic tissue necrosis, causing secondary infections, and increasing the risk of sepsis (Garg and Singh, 2019). The immune response initially aimed at controlling inflammation may become dysregulated, and excessive immune response can lead to the progression of sepsis (Kylänpää et al., 2010). After the occurrence of sepsis, excessive inflammation and immune response are intertwined, manifested as overwhelming systemic infection causing a large amount of inflammatory and uncontrolled immune responses, ultimately leading to multi-organ dysfunction (Mira et al., 2017). The association between SAP and sepsis underscores the complex interaction between inflammation, immunity, and disease progression.

In the past, it was generally believed that pancreatic necrosis in SAP patients was predispose to sepsis, but recent studies have shown that pancreatic injury is an important pathological change in sepsis, which in turn increases the risk of death in patients with SAP-associated sepsis (Li et al., 2020; Chaari et al., 2016). Moreover, SAP combined with sepsis increases mortality rate by 80% (Mifkovic et al., 2006). Therefore, early identification and timely intervention are the key to reduce the risk of SAP-associated sepsis and improve its prognosis.

Although SAP and sepsis share some similar pathophysiological factors, the underlying mechanism of their relationship is unclear, particularly in the setting of cellular and molecular. Inflammation is a typical feature of these two diseases, and the immune state caused by inflammation may affect these two diseases through complex mechanisms, but their immune prospects still require further research. In this work, we identified the shared hub genes and key pathways between SAP and sepsis and preliminarily explored their functions and molecular mechanism using bioinformatics methods. Finally, we assessed the immunological landscape of sepsis and explored the association between shared hub genes with immunocytes to acquire a better knowledge of the pathogenesis of sepsis, which could potentially provide valuable insights for clinical therapeutics.

## 2 Materials and methods

### 2.1 Data collecting

**FIGURE 1:**
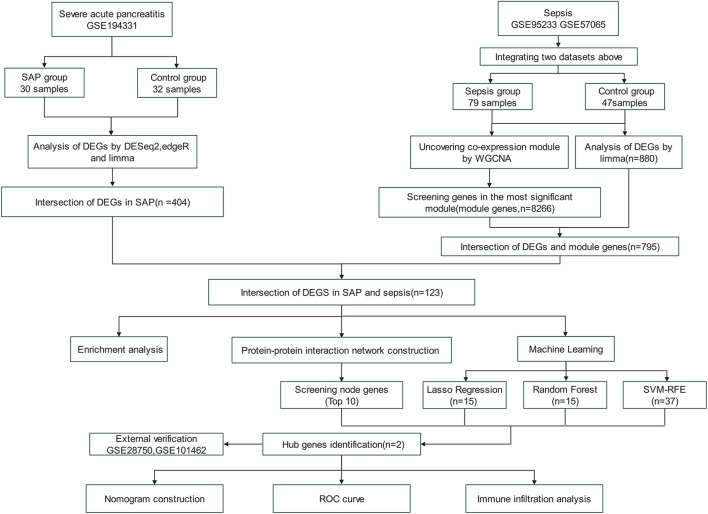
Flow chart of this study.

Figure 1 illustrated the flowchart of the bioinformatics analysis technique employed in this study. The gene expression data for SAP and sepsis was sourced from the GEO database (https://www.ncbi.nlm.nih.gov/geo/) (Barrett et al., 2013). The SAP gene expression dataset GSE194331 based on GPL16791 platform consists of 30 SAP patients and 32 healthy controls. The sepsis microarray dataset GSE95233 comprises 51 sepsis patients and 22 healthy controls, while the GSE57065 collection contains 28 sepsis patients and 25 healthy controls. Both sepsis datasets are based on the GPL570 platform. To ensure maximum consistency and quality, we specifically selected unprocessed blood samples from sepsis patients on the first day as the experimental group for both mentioned datasets. Batch correction was performed on two sepsis datasets by the “removeBatchEffect” function in R software (version 4.2.3) “limma” package, resulting in a combined sepsis expression data consisting of 79 sepsis patients and 47 healthy control samples. Dataset GSE28750 related to sepsis and GSE101462 related to pancreatitis was utilized as the externa validation dataset. Table 1 displays a comprehensive description of the datasets.

**TABLE 1:** Basic information of GEO datasets used in the study.

GSE series	Disease	Origin	Case	Control	Platform	Group
GSE149331	Pancreatitis	PBMC	30	32	GPL16791	Discovery cohort
GSE95233	Sepsis	PBMC	51	22	GPL570	Discovery cohort
GSE57065	Sepsis	PBMC	28	25	GPL570	Discovery cohort
GSE101462	Pancreatitis	PBMC	10	4	GPL10588	Validation cohort
GSE28750	Sepsis	PBMC	10	20	GPL570	Validation cohort

### 2.2 Processing of data and identification of differentially expressed genes (DEGs)

In the GSE194331 high-throughput bulk sequencing dataset, we first extracted the raw gene data. Then, we use R software to read and organize the data. To ensure that the data meets the format requirements of the difference analysis, we need to confirm that the expression matrix is the count data and that the row names correspond to the gene names. Afterwards, we used the “trans_exp_new” function of the “tinyarray” package to convert the ensemble gene names to gene symbols. Subsequently, genes with low expression levels were filtered out, and only those expressed in over 50% of the samples were retained. Finally, we conducted differential gene analysis using the “limma”, “DESeq2”, and “edgeR” package respectively, with a significance threshold set at |log2 Fold change (logFC)| > 1.5 and *P*-value < 0.05 (Ritchie et al., 2015; Love et al., 2014; Robinson et al., 2010).

In the GSE95233 and GSE57065 microarray datasets, we conducted background calibration and normalization using R software. When several probes correspond to the same gene, a random de-weighting method is used, that is, for duplicate probes, only the data of the first probe is retained. As mentioned earlier, the “removeBatchEffect” function was also used to remove batch effects after combining the two sepsis datasets. Finally, we conducted differential gene analysis using the “limma” package using a significance threshold of |logFC| > 1 and *P*-value < 0.05 (Ritchie et al., 2015).

To visualize the expression patterns of DEGs, the “ggplot2” and “pheatmap” packages were conducted to create volcano plots and heatmaps, respectively.

### 2.3 Weighted gene co-expression network analysis (WGCNA)

WGCNA is a biological analysis method used to identify highly collaborative gene sets, which can cluster genes with similar expression patterns and analyze the correlation between modules and specific traits or phenotypes (Langfelder and Horvath, 2008). We first performed hierarchical clustering analysis on all samples to assess the presence of significant outliers. Then, co-expression networks were then constructed using a soft-thresholding approach. We determined the optimal soft threshold power (β = 4) and constructed biologically significant scale-free networks. Subsequently, we established network connectivity using the topology overlap matrix and identified gene modules through the dynamic tree cutting method. After obtaining the gene modules, we calculated the module eigengene based on the first principal component of gene expression profiles within the module and assessed the correlation between module eigengenes and sample traits (disease status). The correlation between module eigengenes and traits represents the correlation between the modules and traits. Finally, we visualized the eigengene network, identified highly correlated modules that are significant in relation to sepsis, and evaluated Module Membership and Gene Significance of the genes in these modules to demonstrate the module significance.

### 2.4 Identification of shared genes and functional enrichment analysis

Initially, we employed a Venn diagram to determine the intersection of SAP DEGs, sepsis DEGs, and sepsis WGCNA key module genes, identifying the overlapping genes as core shared genes (Chen and Boutros, 2011). Subsequently, we conducted functional enrichment analysis on these shared genes. Gene Ontology (GO) provides fundamental annotations and standardized descriptions of gene products, encompassing biological processes, cellular locations, and activities (The Gene Ontology Consortium, 2019). The Kyoto Encyclopedia of Genes and Genomes (KEGG) elucidates significant metabolic pathways, which are particularly crucial in mechanistic research (Ogata et al., 1999). The GO and KEGG enrichment analysis is primarily carried out using the R packages “clusterProfiler”, “enrich”, and “ggplot2”. The statistical difference threshold for GO terms was established as an adjusted *p*-value by Benjamini–Hochberg method of <0.05, while for KEGG pathway, it was set at a *p*-value of <0.05.

### 2.5 Construction of the protein-protein interaction (PPI) network

To investigate the connectivity among shared genes, the Search Tool for Retrieval of Interacting Genes (String) database (https://www.string-db.org) was employed to construct PPI network. The STRING database is an online bioinformatics database that provides information on gene and protein interactions. Using the STRING database, the list of proteins was queried to retrieve potential interactions. Then, the resulting data was used to construct PPI network where nodes represent proteins and edges represent interactions. Subsequently, the PPI network was visualized using the Cytoscape software (version 3.9.1) (Szklarczyk et al., 2021; Shannon et al., 2003). For node ranking based on network features, we primarily employed the CytoHubba plug-in within Cytoscape. 11 topological evaluation approaches are available in CytoHubba, with Maximal Clique Centrality (MCC) exhibiting superior performance in accurately identifying essential proteins from PPI networks (Chin et al., 2014). Therefore, we used the MCC analysis method to select the highest 10 nodes. Finally, the top 10 core genes obtained from the intersection were visualized.

### 2.6 Machine learning

We used three machine learning methods to narrow down the scope of identifying hub genes. The Random Forest (RF) algorithm is designed to reduce variance and improve prediction stability by integrating multiple decision trees. It constructs decision trees using gene subsets and assesses feature importance by the degree to which each gene decreases impurity across the trees (Biau, 2012). The Least absolute shrinkage and selection operator (LASSO), a regression-based method, penalizes coefficients to shrink some gene contributions to zero, effectively performing feature selection by focusing on genes with non-zero coefficients. It is characterized by its ability to increase the accuracy and interpretability of statistical model predictions (Friedman et al., 2010). The Support Vector Machine Recursive Feature Elimination (SVM-RFE) algorithm utilizes structural risk minimization to enhance learning performance by reducing empirical errors while keeping relevant variables. It employs a Support Vector Machine iteratively to rank genes based on their contribution to classification performance, sequentially eliminating the least important features to achieve feature gene selection (Sanz et al., 2018). In the three machine learning models, the dependent variable was the sepsis disease state, and the independent variables were overlapping genes identified through differential analysis and WGCNA of two diseases. Lastly, the common genes obtained by crossing the overlapping genes in the three machine learning models and the top 10 genes of PPI were defined as the hub genes for establishing the diagnostic model of SAP- associated sepsis.

### 2.7 Evaluation of the diagnostic value of hub genes

The nomogram generated by the “rms” R package holds significant value in the clinical diagnosis of SAP-associated sepsis (Mottola and Cocconcelli, 2022). The relative expression level of hub genes translates to a score on the nomogram graph. The total score of the nomogram reflects the aggregation of individual scores, which in turn corresponds to varying diagnostic efficacy for SAP-associated sepsis, and potentially predict its incidence. To assess the diagnostic performance of hub genes for SAP-associated sepsis, we performed receiver operating characteristic (ROC) analysis and utilized the area under the curve (AUC) as the assessment metric. To externally verify our findings, we retrieved the expression data of DEGs from GSE28750 and GSE101462 and performed ROC analysis to evaluate the diagnostic potential value of hub genes.

### 2.8 Immune infiltration analysis

We employed the CIBERSORT method and single sample gene set enrichment analysis (ssGSEA) for immune cell infiltration analysis. CIBERSORT was implemented using the “Cibersort” R package to quantify the distribution of immune cells in sepsis and control groups (Steen et al., 2020). ssGSEA was performed by the “GSVA” R package to excavate the correlation between hub genes and immune cells (Hänzelmann et al., 2013).

## 3 Result

### 3.1 Identification of DEGs in SAP

**FIGURE 2:**
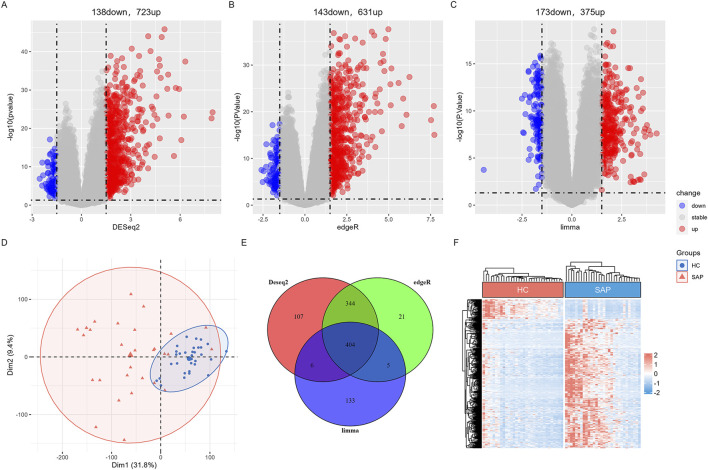
Differential expression gene analysis in SAP dataset **(A)** The volcano map of DEGs by “Deseq2” algorithm. **(B)** The volcano map of DEGs by “edgeR” algorithm. **(C)** The volcano plot of DEGs by “limma” algorithm. **(D)** The distribution characteristics of samples based on PCA results in GSE149331. **(E)** Venn diagram shows that 404 overlapping DEGs in SAP dataset by three algorithms. **(F)** A heatmap of the DEGs in GSE149331.

In the SAP dataset GSE149331, DEGs were screened using the “limma”, “DESeq2”, and “edgeR” package respectively. A total of 404 overlapping genes were classified as SAP DEGs by those three algorithms, including 351 upregulated DEGs and 53 downregulated DEGs. Volcano plots of three algorithms highlighted genes with significant changes and statistical significance, while heatmap depicted the similarity in gene expression between DEGs in SAP dataset (Figures 2A–C, F). PCA plot and the Venn diagram of SAP DEGs of the three analysis algorithms were shown in Figures 2D, E.

### 3.2 Identification of DEGs in sepsis

**FIGURE 3:**
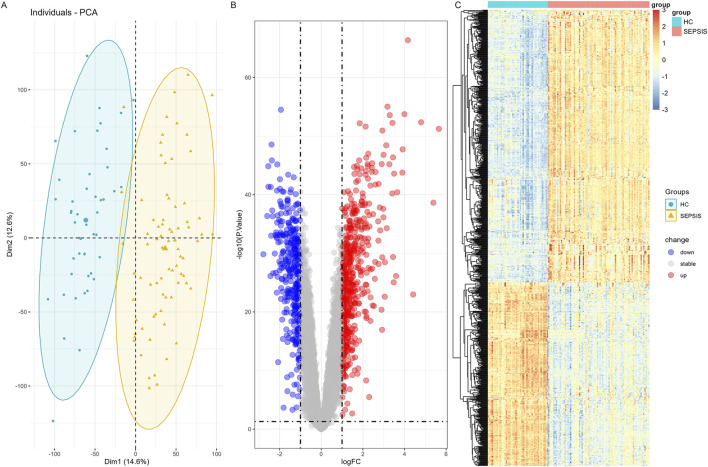
Differential expression gene analysis in sepsis combined dataset **(A)** PCA Plot shows that samples in the sepsis group and control group were clearly distributed on both sides. **(B)** The volcano map of DEGs by “limma” algorithm. **(C)** A heatmap of the DEGs in sepsis combined dataset.

In the sepsis combined dataset, the “limma” algorithm was employed to identify 880 DEGs. Among these, 526 were upregulated while 354 were downregulated. The PCA analysis revealed a significant bilateral distribution of samples between two groups (Figure 3A). The volcano plot (Figure 3B) depicted DEGs expression patterns in sepsis. Figure 3C displayed a cluster heatmap in sepsis dataset.

### 3.3 WGCNA and key module genes screening and shared genes identification

**FIGURE 4:**
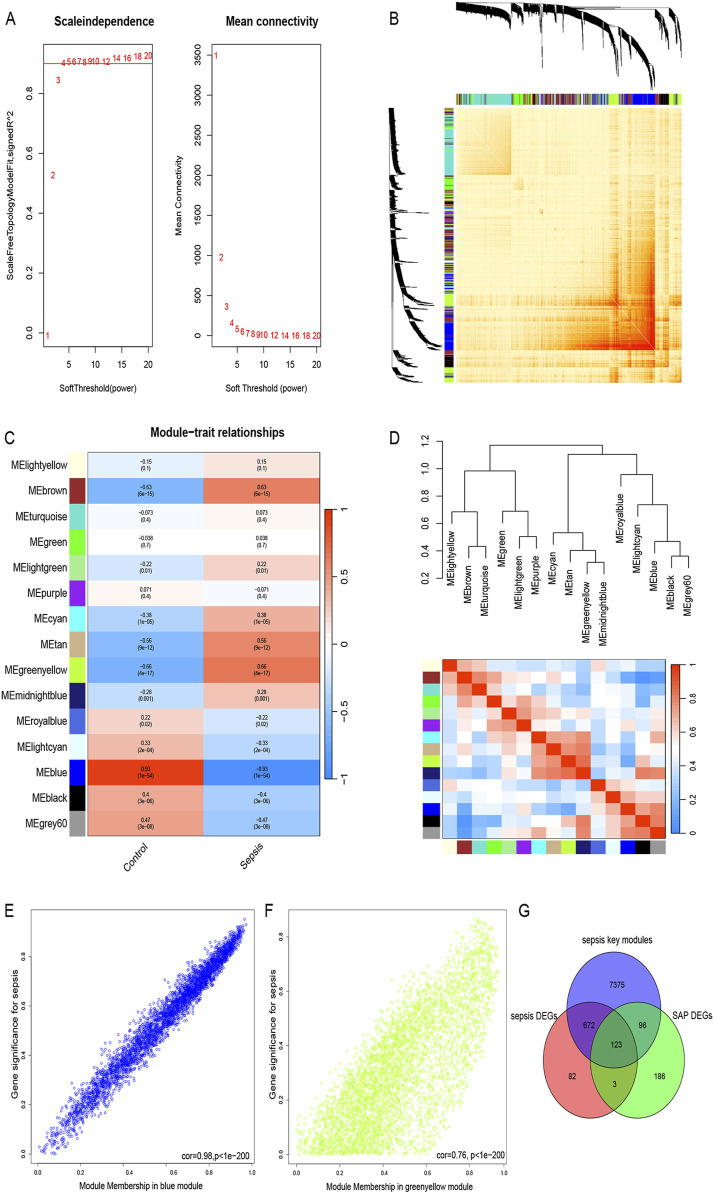
WGCNA, key module genes screening and shared genes identification **(A)** The scale-free topology model was utilized to identify the best β value, and β = 4 was chosen as the soft threshold based on the average connectivity and scale independence. **(B)** The network heatmap showing the gene dendrogram and module eigengenes. **(C)** The heatmap revealing the relationship between module eigengenes and status of sepsis. **(D)** Dendrogram of consensus module eigengenes. **(E)** The correlation plot between the greenyellow Module Membership and the Gene Significance of genes in the greenyellow module. **(F)** The correlation plot between the blue Module Membership and the Gene Significance of genes in the blue module. **(G)** A total of 123 key genes were identified by taking the intersection between two diseases.

WGCNA was performed to excavate the co-expressed gene modules with the highest connectivity and identify the core genes in sepsis. To ensure the connections between genes in the network correspond to a scale-free distribution, we chose a soft-thresholding power β = 4 (scale-free R^2^ = 0.9) and estimated the mean connectivity and scale-free fit index (Figure 4A). Based on this threshold, we divided the genes in the gene clustering tree into modules with high similarity and similar expression patterns, and finally obtained a total of 15 closely related gene modules (Figure 4D). Subsequently, we created a heatmap (Figure 4B) to characterize the Topological Overlap Matrix (TOM) between 1,000 randomly selected genes in the data to visualize the gene network. Furthermore, the relationship between sepsis and gene modules was showed in Figure 4C, the greenyellow module displayed a highly significant positive correlation with sepsis (r = 0.66, *P* < 0.001), whereas the bule module showed the strongest negative correlation with sepsis (r = −0.93, *P* < 0.001). Furthermore, in both greenyellow and blue modules, a noteworthy correlation was observed between Gene Significance and Module Membership (r = 0.76 and r = 0.98, respectively) (Figures 4E, F). Then, a total of 8,266 key genes were obtained in key modules. Finally, we intersected SAP DEGs, sepsis DEGs and core genes of WGCNA in sepsis samples, yielding a total of 123 genes shared by the two diseases (Figure 4G).

### 3.4 Functional enrichment analysis and construction of PPI network

**FIGURE 5:**
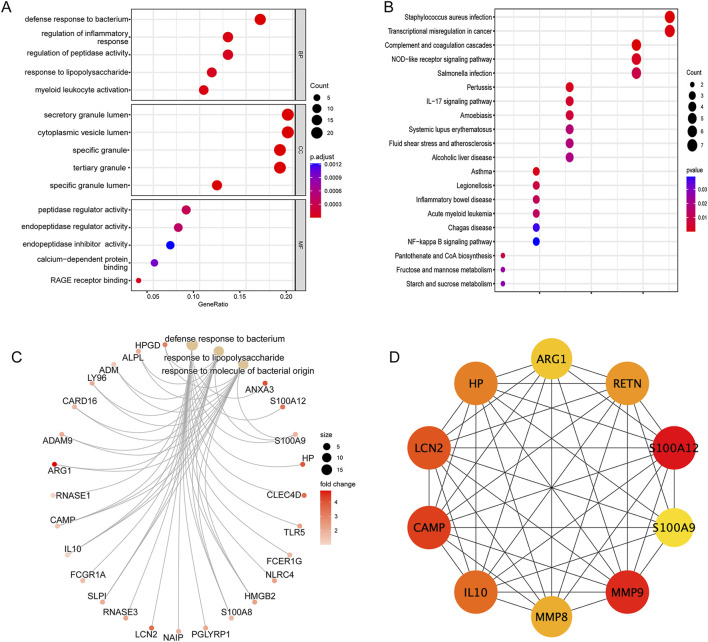
Enrichment analysis of the intersection of genes in sepsis **(A)** GO analysis of the intersection of genes, including biological process, cellular component, and molecular function, respectively. **(B)** KEGG pathway analysis of the intersection of genes. **(C)** The GO cnetplot describes the link between genes and biological concepts as a network and visualize genes involving in enrichment pathways. **(D)** The PPI network of the top 10 core genes based on Cytoscape plug-in CytoHubba analysis.

To explore potential shared biological processes and mechanisms underlying SAP-related sepsis, 123 shared genes were performed GO and KEGG pathways enrichment. Bubble plot was used for visualizing GO analysis, in which three boxes depicted the enrichment analysis outcomes of CC, MF, and BP, respectively. As shown in Figure 5A, GO analysis was substantially enriched in defense response to bacterium, regulation of inflammatory response, regulation of peptidase activity. Furthermore, based on the KEGG analysis (Figure 5B), the shared genes primarily involved in the NF-kappa B (NF-κB) signaling pathway, NOD-like receptor (NLR) signaling pathway, and IL-17 signaling pathway. Cnetplot visualized genes participating in enrichment pathways and displayed the association between genes and biological concepts as a network. The GO cnetplot was adopted to present the gene network under three different terms (Figure 5C).

To evaluate the shared genes interactions between SAP and sepsis, a PPI network of 82 nodes connected by 295 edges was performed by the STRING database. We further visualized the degree of each gene using the CytoHubba plug-in in Cytoscape. Finally, we selected the top 10 most essential genes from the network for further investigation (Figure 5D).

### 3.5 Hub genes screening by machine learning

**FIGURE 6:**
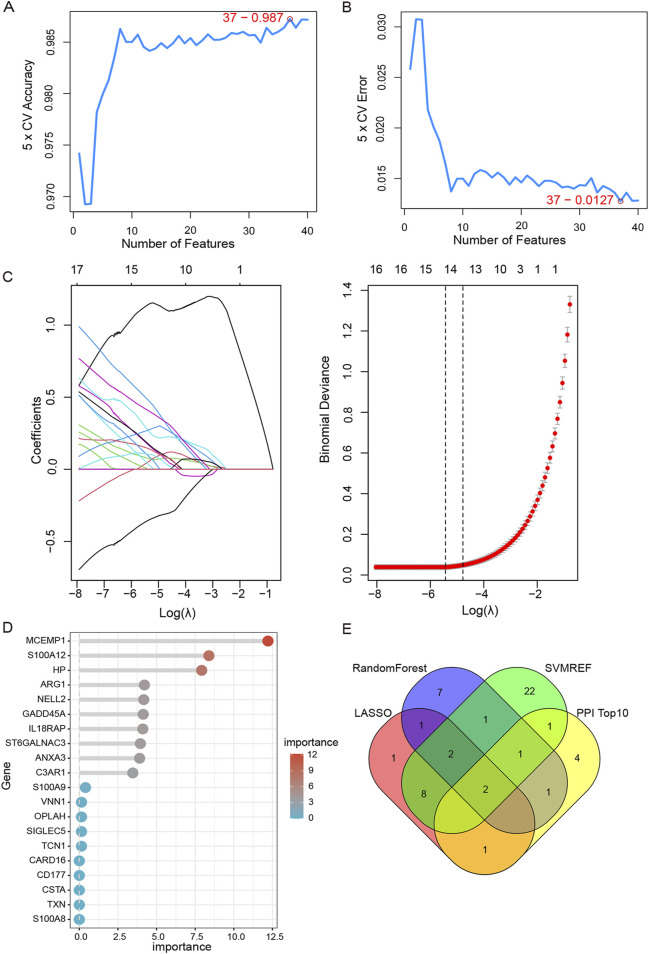
Machine learning and identification of hub genes. **(A,B)** Identification of potential shared diagnostic genes by SVM-RFE model.37 genes were selected based on SVM-RFE with the lowest error and highest accuracy. **(C)** Based on the Lasso regression algorithm, 15 genes were identified as the potential candidate genes with the lowest binominal deviation. **(D)** The top 10 genes were selected and ranked based on the importance score of random forest algorithm. **(E)** Venn diagram shows that two hub genes (ARG1, HP) are identified in SAP- associated sepsis.

To narrow down the scope of screening hub genes, three machine learning methods were adopted. Based on SVM-RFE results, when the feature genes were 37, the classifier achieved maximum precision and minimal error (Figures 6A, B). LASSO regression analysis was performed on 123 shared DEGs, and after tenfold cross validation, the LASSO algorithm identified 15 characteristic genes at the most suitable λ = 0.004 (Figure 6C). The RF algorithm was also carried out to rank 123 shared DEGs according to the importance of each gene. Subsequently, the top 10 characteristic genes with the highest importance were selected (Figure 6D). In the end, by employing a Venn plot to depict the intersection of the overlapping genes of three machine learning algorithms and the top 10 essential genes of PPI, we ultimately identified two hub genes: ARG1 and HP (Figure 6E).

### 3.6 Diagnostic value assessment

**FIGURE 7:**
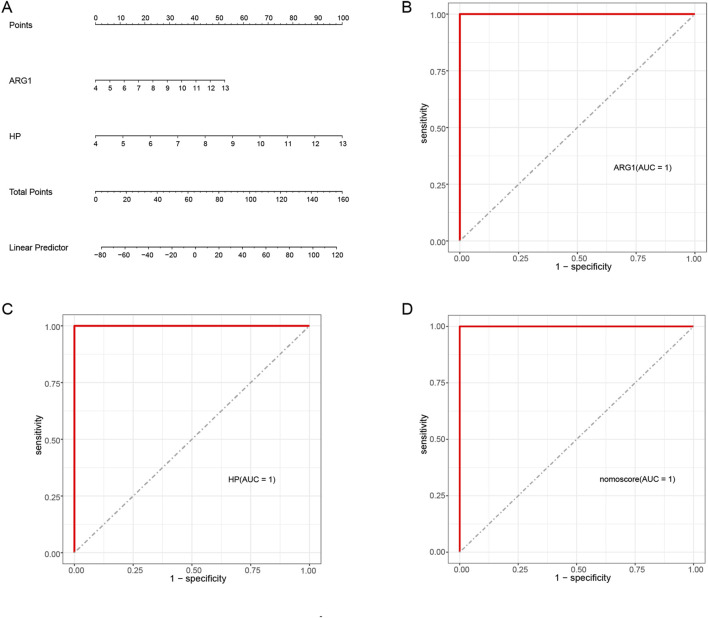
Nomogram construction and the diagnostic value evaluation in SAP-associated sepsis **(A)** The visible nomogram for diagnosing SAP-associated sepsis. **(B)** The ROC curve of ARG1. **(C)** The ROC curve of HP. **(D)** The ROC curve of the nomogram. The ROC curve of each candidate gene and nomogram showed the significant diagnostic value.

To enhance the diagnostic and predictive abilities, a nomogram was established by integrating two hub genes (Figure 7A). To evaluate the sensitivity and specificity of the hub genes and the nomogram in diagnosing SAP-associated sepsis, we calculated their respective AUC values using the ROC curve. Both the two hub genes and the nomogram exhibited an AUC value of 1.000, indicating that the nomogram may have a significant diagnostic utility in SAP-associated sepsis (Figures 7B–D).

**FIGURE 8:**
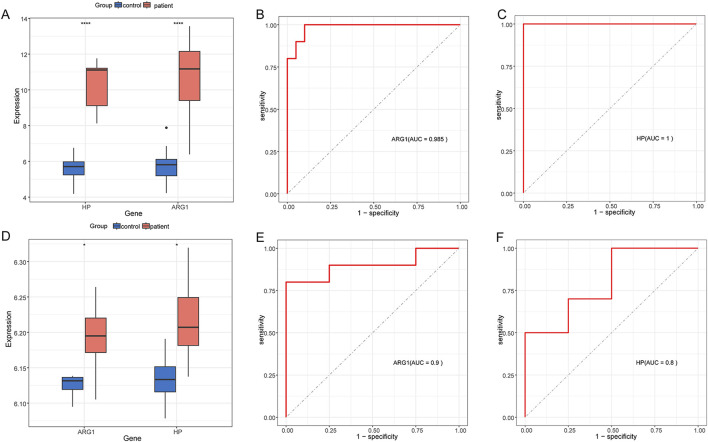
Expression pattern validation and diagnostic value **(A)** Expression of ARG1 and HP in GSE28750. **(B)** ROC curve of the ARG1 in GSE28750. **(C)** ROC curve of the HP in GSE28750. **(D)** Expression of ARG1 and HP in GSE101462. **(E)** ROC curve of the ARG1 in GSE101462. **(F)** ROC curve of the HP in GSE101462. *P < 0.05; ****P < 0.0001.

We conducted external validation of two hub genes to assess their diagnostic performance in GSE28750 and GSE101462 datasets, both of which exhibited effective predictive performance. Figures 8A, D showed the expression patterns of ARG1 and HP in GSE28750 and GSE101462, respectively. ROC analysis of each genes yielded an AUC ≥0.800, demonstrating a good predictive value (Figures 8B, C, E, F).

### 3.7 Immune infiltration analysis

**FIGURE 9:**
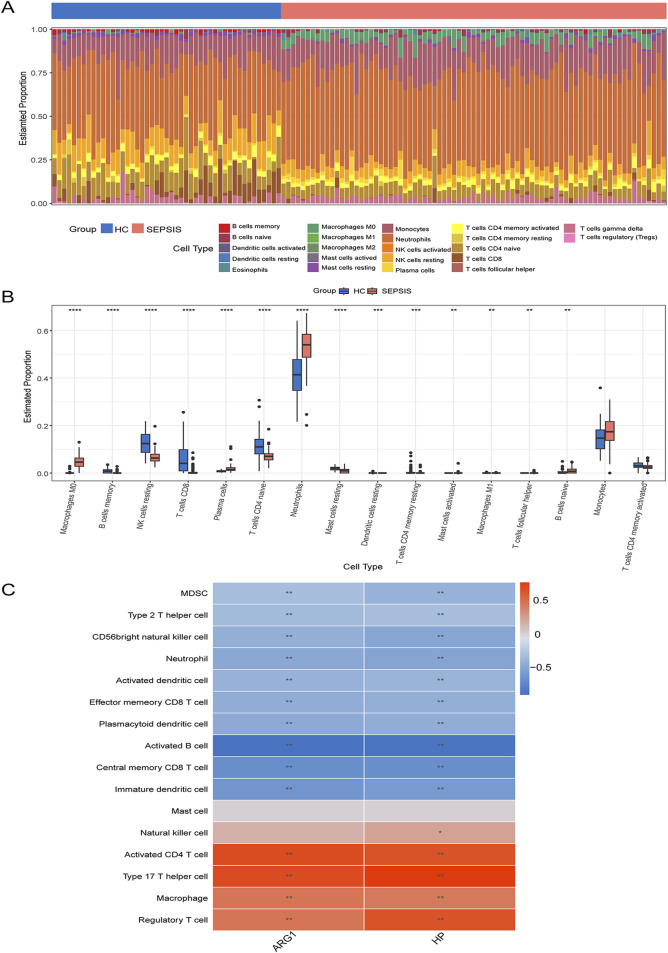
Immune cell infiltration analysis **(A)** The bar plot shows the proportion of immune cells in different samples. **(B)** The boxplot compares the expression of immune cells between sepsis and controls. **(C)** Correlation analysis of immune cell infiltrations with two hub genes. *P < 0.05; **P < 0.01.

To explore the immune landscape in sepsis cohorts, immune cell infiltration was employed by the CIBERSORT and ssGSEA methods. Figure 9A displayed the proportional distribution of 22 different immune cells in the sepsis and control groups. Comparing the sepsis group to the control group, Figure 9B revealed a greater percentage of neutrophils, monocytes, M0 macrophages, naive B cells, and plasma cells, but a lower percentage of resting Natural killer cells, Memory B cells, active memory CD4^+^T cells and CD8^+^T cells. Moreover, ssGSEA was utilized to gain insight into the correlation between two hub genes and the immune cells. Figure 9C demonstrates a significant correlation between both hub genes ARG1 and HP with the multiple immune cells in sepsis.

## 4 Discussion

SAP is a severe pancreatic inflammation characterized by persistent organ damage, often presenting as necrotizing pancreatitis with a considerable morbidity and mortality rate that imposes a social and economic burden (Peery et al., 2022). The course of SAP has two major death peaks, namely, the SIRS or multiple organ failure phase of the first stage (within 2 weeks of onset) and the infection phase of the second stage (within 4 weeks of onset) (Oláh et al., 2007). Sepsis is the usual clinical manifestation at the second stage, characterized by dysregulation of host inflammatory response and multi-organ dysfunction, with a high mortality rate (Mifkovic et al., 2006). Therefore, there is an immediate requirement to explore crucial signaling pathways for a more comprehensive comprehension of regulatory mechanisms and to devise innovative biomarkers that can assist in the timely detection of sepsis associated with SAP.

The SOFA (sequential organ failure assessment) score is a part of the clinical diagnostic criteria for sepsis, which quantifies the criteria for organ damage related to sepsis and can predict the mortality rate of sepsis patients. However, the SOFA score has little reference significance for predicting the occurrence of sepsis (de Grooth et al., 2017). The quick SOFA (qSOFA) score is recommended for screening suspected sepsis, but some studies have indicated that the qSOFA score has poor ability to identify sepsis and predict death (Fernando et al., 2018; Hwang et al., 2018). Procalcitonin (PCT) is considered a promising biomarker that can be used to guide the use of antibiotics, but its non-specific nature limits its diagnostic efficacy in clinical applications (Nobre et al., 2008). In recent years, research on novel biomarkers for sepsis has been constantly emerging, but so far, none of them have been strongly proven to be superior (Pierrakos et al., 2020). Previously, there was no research combining SAP and sepsis to determine diagnostic biomarkers for SAP associated sepsis and establish a diagnostic model. For the first time in this research, we have integrated SAP and sepsis transcriptome and identified hub genes linked to SAP and sepsis. Furthermore, we have analyzed their shared regulatory pathways, immune landscape, and clinical implications. We found that ARG1 and HP are the most important hub genes between SAP and sepsis, and the NLR signaling pathway is a common regulatory pathway for these two diseases. Finally, ARG1 and HP were identified as valuable diagnostic markers and nomogram maps were developed to improve early identification of SAP-associated sepsis. The results of immune infiltration indicated that the two hub genes were significantly correlated with immune cells and may serve as therapeutic targets for SAP-associated sepsis.

The enrichment analyses confirmed that the hub genes between SAP and sepsis were involved in the NLR signaling pathway. NLR, a family member of pathogen recognition receptor, is critical in intracellular ligand recognition (Platnich and Muruve, 2019). NOD1 and NOD2, two typical NLRs, can be activated by specific components of bacterial peptidoglycans. Once activated, it may trigger the downstream NF-κB pathway, resulting in the secretion of inflammatory chemokines (Caruso et al., 2014). The NOD-like receptor family pyrin domain-containing 3 (NLRP3) inflammasome, which belongs to the NLR family, has also been proven to be linked to the initiation and progression of SAP and sepsis (Danielski et al., 2020; Ferrero-Andrés et al., 2020). In the context of sepsis, NLRP3 functions by recruiting and activating caspase-1, leading to release of IL-1β and IL-18, initiating a cascade of inflammatory reactions that ultimately leads to substantial inflammation-induced damage and multi-organ failure (Sahoo et al., 2011). Furthermore, caspase-1 mediated immune cell pyroptosis may lead to immune paralysis in sepsis (Miao et al., 2011). In another study, activation of the NLRP3 inflammasome was observed in SAP animal models, and downregulating this pathway led to alleviation of intestinal injury (Xu et al., 2018). Collectively, the NLR signaling pathway contributes significantly to the shared pathophysiology of these two diseases. We speculate that the inflammation and immune processes mediated by the NLR signaling pathway are potential mechanisms for the occurrence and development of SAP-associated sepsis. Targeted downregulation of the NLR signaling pathway and inhibition of inflammasome activation may be a promising treatment strategy for SAP-associated sepsis.

Our research indicates that both ARG1 and HP were upregulated in SAP and sepsis and showed good predictive properties with an AUC ≥0.800 in both test and validation sets. Additionally, our study developed a nomogram for diagnosing SAP-associated sepsis for the first time, which has higher diagnostic value than independent biomarkers. We can collect peripheral blood from SAP patients and infer the probability of sepsis occurrence based on the expression of two hub genes. Our diagnostic nomogram incorporates critical insights into the NLR signaling pathway, particularly through the hub genes ARG1 and HP, which bridge SAP and sepsis. These genes are an indispensable part of immune and inflammatory regulation, and are enriched in the NLR signaling pathway, which is a common regulatory mechanism of the two diseases. By focusing on gene expression levels and pathway activity indicators related to NLR signaling, our model captures the dynamic immune response in SAP-associated sepsis, improving our understanding of the disease pathogenesis. This model represents a significant advancement in SAP and sepsis management, providing a shift from passive to active patient treatment. The early prediction of the model can not only guide us in early monitoring and intervention of SAP patients, prevent SAP from developing into sepsis, reduce the probability of sepsis occurrence, but also improve the timeliness and effectiveness of treatment interventions, optimize patient management strategies, prevent sepsis from developing to more severe stages, thereby reducing mortality and improving clinical outcomes.

As an important hub gene between SAP and sepsis, ARG1 (Arginase 1) is a member of the urea hydrolase family and participates in the metabolism of arginine by catalyzing the hydrolysis of arginine into urea and ornithine (Wissmann et al., 1996). More and more evidence confirm that arginase activity is upregulated during SAP and sepsis (Ding et al., 2022; Reizine et al., 2022; Chu et al., 2021). A study has found an increase in the expression and activity of ARG1 in patients with SAP (Ding et al., 2022). In addition, the expression of ARG1 in neutrophils is significantly upregulated in sepsis patients, and inhibition of ARG1 can significantly restore the immune function of CD8^+^T cells (Dai et al., 2022). The evidence presented above indicates that it may be related to the development and progression of SAP and sepsis. Arginase has been shown to inhibit T cell function by downregulating the expression of T cell receptors, and supplementing with L-arginine can promote T cell survival in the absence of external cytokines, highlighting the key function of ARG1 in immune suppression (Geiger et al., 2016). In addition, arginase competes with nitric oxide synthase for arginine as a substrate, interfering with carbon monoxide synthase activity and leading to vascular dysfunction (Hu et al., 2021). ARG1 has always been considered a classic biomarker in M2 macrophages. It participates in the antibacterial pathway of polymorphonuclear granulocytes and can regulate macrophage function by metabolizing arginine, leading to sustained and aggravated inflammatory responses (Yang and Ming, 2014). We speculate that ARG1 may participate in the pathological and physiological processes of these two diseases through the above mechanisms, and arginine metabolism may be a potential target for controlling infection and immunity in SAP-associated sepsis.

HP (Haptoglobin) is a protein mainly produced in the liver, responsible for binding and clearing free hemoglobin. Additionally, HP is also an acute phase protein and exhibits antimicrobial properties (Eaton et al., 1982). Its association with inflammatory diseases is widely recognized (Quaye, 2008). Previous reports have shown that HP levels increase in both pediatric and adult with sepsis (Chavez-Bueno et al., 2011; Philip, 2012; Thongboonkerd et al., 2009). HP levels have been utilized in algorithms to assist in the diagnosis of sepsis (Philip, 2012). However, there is relatively little research on haptoglobin in acute pancreatitis. Only one animal experiment published in 2020 was detected, which showed an increase in haptoglobin levels observed during the acute phase of acute pancreatitis (Yoon et al., 2020). Research has found that β Chains of HP can bind to the adhesion glycoprotein CD22 of B lymphocytes, thereby having an inhibitory effect on the function of B lymphocytes (Hanasaki et al., 1995). HP can also exert immune regulatory effects by affecting T cell function and regulating macrophage polarization (Arredouani et al., 2003; Morimoto et al., 2022). We assumed that HP may play a potential role in SAP and sepsis by affecting the intensity of inflammatory response and activation status of immune cells, but further data is needed.

ARG1 and HP play different but complementary roles in the pathophysiology of SAP- associated sepsis. ARG1 mainly reflects immune suppression, while HP mainly reflects inflammatory status. Their combined analysis can comprehensively evaluate immune function and inflammatory status, improve the overall predictive ability and clinical utility of the model. Given the role of ARG1 in immune regulation, ARG1 levels can provide insights into the immune status and potential prognosis of patients. A higher level of ARG1 may indicate severe immune suppression, indicating a higher risk of adverse consequences. Meanwhile, understanding ARG1 dynamics can also help identify potential therapeutic targets. By using ARG1 as an important feature gene in the model, the model can predict which patients may benefit from treatments aimed at regulating arginine metabolism or enhancing immune response. HP serves as a valuable marker of inflammatory status, distinguishing different stages, and types of inflammatory reactions in SAP-associated sepsis. Incorporating HP levels into the model helps identify high-risk patients who may require more proactive intervention in the early stages. Utilizing both ARG1 and HP enables the model to achieve more personalized treatment. Treatment strategies based on ARG1 and HP levels can more effectively address specific pathophysiological mechanisms, providing patients with precise medical services and improving prognosis.

Our immune infiltration analysis suggests that various types of immune cells are involved in the occurrence and development of sepsis. Immune cells are pivotal in orchestrating the immune response and preserving immune homeostasis during sepsis. Understanding the changes of different immune cell populations in sepsis and analyzing the quantity, functional status, and interactions of different immune cell populations can help us gain a deeper understanding of the development mechanism and predict the effectiveness of treatment, which may bring new ideas for the treatment of sepsis. In the initial stage of sepsis, neutrophils serve as the foremost defensive mechanism against sepsis by virtue of their rapid migration to the infection site (Tan et al., 2020). Macrophages polarize towards M1 macrophages and initiate a pro-inflammatory cascade with neutrophils, releasing a substantial amount of inflammatory and chemotactic factors (Cavaillon and Adib-Conquy, 2005). In addition, the number and function of B cells and T lymphocytes have significantly decreased, including a decrease in antigen presentation ability and a decrease in lymphocyte proliferation activity (Wiersinga and van der Poll, 2022). Our research results indicate notable variations in immunological cell profiles between the sepsis and control groups. The sepsis group showed an elevation in monocytes, macrophages, and neutrophils, alongside a decrease in Memory B cell, CD4^+^T cells, and CD8^+^T cells. ARG1 and HP, identified as crucial hub genes between SAP and sepsis, play a vital role in the immunological response. Our study found that in sepsis samples, two key genes are positively correlated with macrophages and regulatory T cells and negatively correlated with activated B cells and CD8^+^T cells. This will shed light on immune-level pathophysiological mechanisms and may be an essential factor in comprehending the pathogenesis of SAP-associated sepsis.

Our research has several advantages. We used comprehensive bioinformatics analysis for the first time to understand the relationship between these two diseases. WGCNA and three machine learning algorithms were used to identify potential shared diagnostic genes, and a nomogram for diagnosing SAP- associated sepsis was developed for the first time. The validation of external datasets improved the accuracy of predictions. There are also three major limitations in this study. Firstly, the data used in this study was sourced from public databases. Public databases often have sample bias and the compilation of data from different sources in public databases leads to inconsistent data quality. However, in our study, we have expanded the data by integrating different sepsis datasets to balance the representativeness of different subgroups and reduce selection bias. At the same time, we applied strict data preprocessing and standardization techniques when processing data to alleviate quality inconsistencies caused by different data collections. Secondly, although we expanded the sample size by merging two sepsis datasets and using an external validation set to reduce the risk of overfitting, our study sample size may still be valid, and the AUC obtained from the experimental results is slightly perfect, indicating the possibility of overfitting. Finally, our research findings come from different patient cohorts and have not yet been validated within the same individual. Therefore, it is necessary to develop an SAP-sepsis combination model, integrate available clinical metadata into the model as much as possible, and conduct extensive functional validation in large-scale studies with sufficient sample size.

## 5 Conclusion

ARG1 and HP may affect SAP and sepsis by regulating inflammation and immune responses, shedding light on potential future diagnostic and therapeutic approaches for SAP-associated sepsis.

## Data Availability

The datasets presented in this study can be found in online repositories. The names of the repository/repositories and accession number(s) can be found in the article/supplementary material.

## Abbreviations

AP, Acute pancreatitis; SAP, Severe acute pancreatitis; GEO, Gene Expression Omnibus; FC, Fold change; WGCNA, Weighted gene co-expression network analysis; DEG, Differentially expressed gene; PPI, Protein–protein interaction; RF, Random Forest; LASSO, Least absolute shrinkage and selection operator; SVM-RFE, Support vector machine recursive feature elimination; ROC, Receiver operating characteristic; AUC, area under the curve; SsGSEA, single sample gene set enrichment analysis; GO, Gene Ontology; KEGG, Kyoto Encyclopedia of Genes and Genomes; NLR, NOD-like receptor; NF-κB, NF-kappa B; SIRS, systemic inflammatory response syndrome; SOFA, sequential organ failure assessment; qSOFA, quick SOFA; PCT, Procalcitonin; NLRP3, NOD-like receptor family pyrin domain-containing 3; ARG1, Arginase 1; HP, Haptoglobin.

## References

[B1] Arredouani M. Matthijs P. Van Hoeyveld E. Kasran A. Baumann H. Ceuppens J. L. (2003). Haptoglobin directly affects T cells and suppresses T helper cell type 2 cytokine release. Immunology 108 (2), 144–151. 10.1046/j.1365-2567.2003.01569.x 12562322 PMC1782886

[B2] Barrett T. Wilhite S. E. Ledoux P. Evangelista C. Kim I. F. Tomashevsky M. (2013). NCBI GEO: archive for functional genomics data sets--update. Nucleic Acids Res. 41 (Database issue), D991–D995. 10.1093/nar/gks1193 23193258 PMC3531084

[B3] Beger H. G. Rau B. M. (2007). Severe acute pancreatitis: clinical course and management. World J. Gastroenterol. 13 (38), 5043–5051. 10.3748/wjg.v13.i38.5043 17876868 PMC4434632

[B4] Biau G. (2012). Analysis of a random forests model. J. Mach. Learn. Res. 13, 1063–1095. 10.5555/2188385.2343682

[B5] Boxhoorn L. Voermans R. P. Bouwense S. A. Bruno M. J. Verdonk R. C. Boermeester M. A. (2020). Acute pancreatitis. Lancet 396 (10252), 726–734. 10.1016/S0140-6736(20)31310-6 32891214

[B6] Caruso R. Warner N. Inohara N. Núñez G. (2014). NOD1 and NOD2: signaling, host defense, and inflammatory disease. Immunity 41 (6), 898–908. 10.1016/j.immuni.2014.12.010 25526305 PMC4272446

[B7] Cavaillon J. M. Adib-Conquy M. (2005). Monocytes/macrophages and sepsis. Crit. Care Med. 33 (12 Suppl. l), S506–S509. 10.1097/01.ccm.0000185502.21012.37 16340435

[B8] Chaari A. Abdel Hakim K. Bousselmi K. Etman M. El Bahr M. El Saka A. (2016). Pancreatic injury in patients with septic shock: a literature review. World J. Gastrointest. Oncol. 8 (7), 526–531. 10.4251/wjgo.v8.i7.526 27559431 PMC4942740

[B9] Chavez-Bueno S. Beasley J. A. Goldbeck J. M. Bright B. C. Morton D. J. Whitby P. W. (2011). Haptoglobin concentrations in preterm and term newborns. J. Perinatol. 31 (7), 500–503. 10.1038/jp.2010.197 21252963

[B10] Chen H. Boutros P. C. (2011). VennDiagram: a package for the generation of highly-customizable Venn and Euler diagrams in R. BMC Bioinforma. 12, 35. 10.1186/1471-2105-12-35 PMC3041657 21269502

[B11] Chin C. H. Chen S. H. Wu H. H. Ho C. W. Ko M. T. Lin C. Y. (2014). cytoHubba: identifying hub objects and sub-networks from complex interactome. BMC Syst. Biol. 8 (Suppl. 4), S11. 10.1186/1752-0509-8-S4-S11 25521941 PMC4290687

[B12] Chu X. Di C. Chang P. Li L. Feng Z. Xiao S. (2021). Lactylated histone H3K18 as a potential biomarker for the diagnosis and predicting the severity of septic shock. Front. Immunol. 12, 786666. 10.3389/fimmu.2021.786666 35069560 PMC8773995

[B13] Dai X. K. Ding Z. X. Tan Y. Y. Bao H. R. Wang D. Y. Zhang H. (2022). Neutrophils inhibit CD8(+) T cells immune response by arginase-1 signaling in patients with sepsis. World J. Emerg. Med. 13 (4), 266–273. 10.5847/wjem.j.1920-8642.2022.068 35837557 PMC9233973

[B14] Danielski L. G. Giustina A. D. Bonfante S. Barichello T. Petronilho F. (2020). The NLRP3 inflammasome and its role in sepsis development. Inflammation 43 (1), 24–31. 10.1007/s10753-019-01124-9 31741197

[B15] de Grooth H. J. Geenen I. L. Girbes A. R. Vincent J. L. Parienti J. J. Oudemans-van Straaten H. M. (2017). SOFA and mortality endpoints in randomized controlled trials: a systematic review and meta-regression analysis. Crit. Care 21 (1), 38. 10.1186/s13054-017-1609-1 28231816 PMC5324238

[B16] Ding L. Wan M. Wang D. Cao H. Wang H. Gao P. (2022). Myeloid-derived suppressor cells in patients with acute pancreatitis with increased inhibitory function. Front. Immunol. 13, 840620. 10.3389/fimmu.2022.840620 35911709 PMC9329796

[B17] Eaton J. W. Brandt P. Mahoney J. R. Lee J. T. Jr (1982). Haptoglobin: a natural bacteriostat. Science 215 (4533), 691–693. 10.1126/science.7036344 7036344

[B18] Fernando S. M. Tran A. Taljaard M. Cheng W. Perry J. J. (2018). Prognostic accuracy of the quick sequential organ failure assessment for mortality in patients with suspected infection. Ann. Intern Med. 169 (4), 264–265. 10.7326/L18-0290 30128520

[B19] Ferrero-Andrés A. Panisello-Roselló A. Roselló-Catafau J. Folch-Puy E. (2020). NLRP3 inflammasome-mediated inflammation in acute pancreatitis. Int. J. Mol. Sci. 21 (15), 5386. 10.3390/ijms21155386 32751171 PMC7432368

[B20] Friedman J. Hastie T. Tibshirani R. (2010). Regularization paths for generalized linear models via coordinate descent. J. Stat. Softw. 33 (1), 1–22. 10.18637/jss.v033.i01 20808728 PMC2929880

[B21] Garg P. K. Singh V. P. (2019). Organ failure due to systemic injury in acute pancreatitis. Gastroenterology 156 (7), 2008–2023. 10.1053/j.gastro.2018.12.041 30768987 PMC6486861

[B22] Geiger R. Rieckmann J. C. Wolf T. Basso C. Feng Y. Fuhrer T. (2016). L-arginine modulates T cell metabolism and enhances survival and anti-tumor activity. Cell. 167 (3), 829–842. 10.1016/j.cell.2016.09.031 27745970 PMC5075284

[B23] Hanasaki K. Powell L. D. Varki A. (1995). Binding of human plasma sialoglycoproteins by the B cell-specific lectin CD22. Selective recognition of immunoglobulin M and haptoglobin. J. Biol. Chem. 270 (13), 7543–7550. 10.1074/jbc.270.13.7543 7706301

[B24] Hänzelmann S. Castelo R. Guinney J. (2013). GSVA: gene set variation analysis for microarray and RNA-seq data. BMC Bioinforma. 14, 7. 10.1186/1471-2105-14-7 PMC3618321 23323831

[B25] Hu S. Pi Q. Xu X. Yan J. Guo Y. Tan W. (2021). Disrupted eNOS activity and expression account for vasodilator dysfunction in different stage of sepsis. Life Sci. 264, 118606. 10.1016/j.lfs.2020.118606 33091444

[B26] Hwang S. Y. Jo I. J. Lee S. U. Lee T. R. Yoon H. Cha W. C. (2018). Low accuracy of positive qSOFA criteria for predicting 28-day mortality in critically ill septic patients during the early period after emergency department presentation. Ann. Emerg. Med. 71 (1), 1–9. 10.1016/j.annemergmed.2017.05.022 28669551

[B27] Kylänpää L. Rakonczay Z. Jr. O'Reilly D. A. (2012). The clinical course of acute pancreatitis and the inflammatory mediators that drive it. Int. J. Inflam. 2012, 360685. 10.1155/2012/360685 23304633 PMC3530799

[B28] Kylänpää M. L. Repo H. Puolakkainen P. A. (2010). Inflammation and immunosuppression in severe acute pancreatitis. World J. Gastroenterol. 16 (23), 2867–2872. 10.3748/wjg.v16.i23.2867 20556831 PMC2887581

[B29] Langfelder P. Horvath S. (2008). WGCNA: an R package for weighted correlation network analysis. BMC Bioinforma. 9, 559. 10.1186/1471-2105-9-559 PMC2631488 19114008

[B30] Lee P. J. Papachristou G. I. (2019). New insights into acute pancreatitis. Nat. Rev. Gastroenterol. Hepatol. 16 (8), 479–496. 10.1038/s41575-019-0158-2 31138897

[B31] Li Y. Suo L. Zhang J. (2020). Role of autophagy in pancreatic injury in sepsis. Zhonghua Wei Zhong Bing Ji Jiu Yi Xue 32 (4), 504–507. 10.3760/cma.j.cn121430-20191021-00063 32527363

[B32] Love M. I. Huber W. Anders S. (2014). Moderated estimation of fold change and dispersion for RNA-seq data with DESeq2. Genome Biol. 15 (12), 550. 10.1186/s13059-014-0550-8 25516281 PMC4302049

[B33] Miao E. A. Rajan J. V. Aderem A. (2011). Caspase-1-induced pyroptotic cell death. Immunol. Rev. 243 (1), 206–214. 10.1111/j.1600-065X.2011.01044.x 21884178 PMC3609431

[B34] Mifkovic A. Pindak D. Daniel I. Pechan J. (2006). Septic complications of acute pancreatitis. Bratisl. Lek. Listy 107 (8), 296–313. 17125065

[B35] Mira J. C. Gentile L. F. Mathias B. J. Efron P. A. Brakenridge S. C. Mohr A. M. (2017). Sepsis pathophysiology, chronic critical illness, and persistent inflammation-immunosuppression and catabolism syndrome. Crit. Care Med. 45 (2), 253–262. 10.1097/CCM.0000000000002074 27632674 PMC5243156

[B36] Morimoto M. Nakano T. Egashira S. Irie K. Matsuyama K. Wada M. (2022). Haptoglobin regulates macrophage/microglia-induced inflammation and prevents ischemic brain damage via binding to HMGB1. J. Am. Heart Assoc. 11 (6), e024424. 10.1161/JAHA.121.024424 35243897 PMC9075294

[B37] Mottola G. Cocconcelli M. (2022). Nomograms: an old Tool with new applications. 7th international symposium on history of machines and mechanisms (HMM) (Spain: Univ Jaen). 10.1007/978-3-030-98499-1_26

[B38] Nobre V. Harbarth S. Graf J. D. Rohner P. Pugin J. (2008). Use of procalcitonin to shorten antibiotic treatment duration in septic patients: a randomized trial. Am. J. Respir. Crit. Care Med. 177 (5), 498–505. 10.1164/rccm.200708-1238OC 18096708

[B39] Ogata H. Goto S. Sato K. Fujibuchi W. Bono H. Kanehisa M. (1999). KEGG: Kyoto Encyclopedia of genes and Genomes. Nucleic Acids Res. 27 (1), 29–34. 10.1093/nar/27.1.29 9847135 PMC148090

[B40] Oláh A. Pardavi G. Belágyi T. Romics L. Jr (2007). Preventive strategies for septic complications of acute pancreatitis. Chir. (Bucur) 102 (4), 383–388. 17966933

[B41] Peery A. F. Crockett S. D. Murphy C. C. Jensen E. T. Kim H. P. Egberg M. D. (2022). Burden and cost of gastrointestinal, liver, and pancreatic diseases in the United States: update 2021. Gastroenterology 162 (2), 621–644. 10.1053/j.gastro.2021.10.017 34678215 PMC10756322

[B42] Philip A. G. (2012). Haptoglobin in diagnosis of sepsis. J. Perinatol. 32 (4), 312. author reply 3. 10.1038/jp.2011.189 22460602

[B43] Pierrakos C. Velissaris D. Bisdorff M. Marshall J. C. Vincent J. L. (2020). Biomarkers of sepsis: time for a reappraisal. Crit. Care 24 (1), 287. 10.1186/s13054-020-02993-5 32503670 PMC7273821

[B44] Platnich J. M. Muruve D. A. (2019). NOD-like receptors and inflammasomes: a review of their canonical and non-canonical signaling pathways. Arch. Biochem. Biophys. 670, 4–14. 10.1016/j.abb.2019.02.008 30772258

[B45] Quaye I. K. (2008). Haptoglobin, inflammation and disease. Trans. R. Soc. Trop. Med. Hyg. 102 (8), 735–742. 10.1016/j.trstmh.2008.04.010 18486167

[B46] Reizine F. Grégoire M. Lesouhaitier M. Coirier V. Gauthier J. Delaloy C. (2022). Beneficial effects of citrulline enteral administration on sepsis-induced T cell mitochondrial dysfunction. Proc. Natl. Acad. Sci. U. S. A. 119 (8), e2115139119. 10.1073/pnas.2115139119 35173051 PMC8872724

[B47] Ritchie M. E. Phipson B. Wu D. Hu Y. Law C. W. Shi W. (2015). Limma powers differential expression analyses for RNA-sequencing and microarray studies. Nucleic Acids Res. 43 (7), e47. 10.1093/nar/gkv007 25605792 PMC4402510

[B48] Robinson M. D. McCarthy D. J. Smyth G. K. (2010). edgeR: a Bioconductor package for differential expression analysis of digital gene expression data. Bioinformatics 26 (1), 139–140. 10.1093/bioinformatics/btp616 19910308 PMC2796818

[B49] Sahoo M. Ceballos-Olvera I. del Barrio L. Re F. (2011). Role of the inflammasome, IL-1β, and IL-18 in bacterial infections. ScientificWorldJournal 11, 2037–2050. 10.1100/2011/212680 22125454 PMC3217589

[B50] Sanz H. Valim C. Vegas E. Oller J. M. Reverter F. (2018). SVM-RFE: selection and visualization of the most relevant features through non-linear kernels. Bmc Bioinforma. 19, 432. 10.1186/s12859-018-2451-4 PMC6245920 30453885

[B51] Shannon P. Markiel A. Ozier O. Baliga N. S. Wang J. T. Ramage D. (2003). Cytoscape: a software environment for integrated models of biomolecular interaction networks. Genome Res. 13 (11), 2498–2504. 10.1101/gr.1239303 14597658 PMC403769

[B52] Singer M. Deutschman C. S. Seymour C. W. Shankar-Hari M. Annane D. Bauer M. (2016). The third international consensus definitions for sepsis and septic shock (Sepsis-3). Jama 315 (8), 801–810. 10.1001/jama.2016.0287 26903338 PMC4968574

[B53] Steen C. B. Liu C. L. Alizadeh A. A. Newman A. M. (2020). Profiling cell type abundance and expression in bulk tissues with CIBERSORTx. Methods Mol. Biol. 2117, 135–157. 10.1007/978-1-0716-0301-7_7 31960376 PMC7695353

[B54] Szklarczyk D. Gable A. L. Nastou K. C. Lyon D. Kirsch R. Pyysalo S. (2021). The STRING database in 2021: customizable protein-protein networks, and functional characterization of user-uploaded gene/measurement sets. Nucleic Acids Res. 49 (D1), D605–D612. 10.1093/nar/gkaa1074 33237311 PMC7779004

[B55] Tan C. Gu J. Chen H. Li T. Deng H. Liu K. (2020). Inhibition of aerobic glycolysis promotes neutrophil to influx to the infectious site via CXCR2 in sepsis. Shock 53 (1), 114–123. 10.1097/SHK.0000000000001334 30829852

[B56] The Gene Ontology Consortium (2019). The gene Ontology resource: 20 years and still GOing strong. Nucleic Acids Res. 47 (D1), D330–D338. 10.1093/nar/gky1055 30395331 PMC6323945

[B57] Thongboonkerd V. Chiangjong W. Mares J. Moravec J. Tuma Z. Karvunidis T. (2009). Altered plasma proteome during an early phase of peritonitis-induced sepsis. Clin. Sci. (Lond). 116 (9), 721–730. 10.1042/CS20080478 19006484

[B58] Wiersinga W. J. van der Poll T. (2022). Immunopathophysiology of human sepsis. EBioMedicine 86, 104363. 10.1016/j.ebiom.2022.104363 36470832 PMC9783164

[B59] Wissmann P. B. Goodman B. K. Vockley J. G. Kern R. M. Cederbaum S. D. Grody W. W. (1996). Delivery of cytosolic liver arginase into the mitochondrial matrix space: a possible novel site for gene replacement therapy. Somat. Cell. Mol. Genet. 22 (6), 489–498. 10.1007/BF02369440 9131018

[B60] Xu S. Wei S. Guo Y. Cui D. Yao J. (2018). Involvement of nucleotide-binding and oligomerization domain-like receptors in the intestinal injury of severe acute pancreatitis in rats. Pancreas 47 (2), 245–251. 10.1097/MPA.0000000000000977 29303910

[B61] Yang Z. Ming X. F. (2014). Functions of arginase isoforms in macrophage inflammatory responses: impact on cardiovascular diseases and metabolic disorders. Front. Immunol. 5, 533. 10.3389/fimmu.2014.00533 25386179 PMC4209887

[B62] Yoon J. S. Kim S. Kang J. H. Park J. Yu D. (2020). Alterations in serum protein electrophoresis profiles during the acute phase response in dogs with acute pancreatitis. Can. J. Vet. Res. 84 (1), 74–78. 31949331 PMC6923814

